# PPAR-*γ* Activation Exerts an Anti-inflammatory Effect by Suppressing the NLRP3 Inflammasome in Spinal Cord-Derived Neurons

**DOI:** 10.1155/2019/6386729

**Published:** 2019-03-13

**Authors:** Qing-Qi Meng, Zhen-Cheng Feng, Xing-Liang Zhang, Li-Qiong Hu, Min Wang, Hai-Feng Zhang, Si-Ming Li

**Affiliations:** ^1^Department of Orthopedics of Guangzhou Red Cross Hospital, Medical College, Jinan University, Tongfu Road 396, 107, 510120 Guangzhou, China; ^2^Shenzhen Children's Hospital, Yitian Road 1079, Futian District, 518038 Shenzhen, China; ^3^Department of Intensive Care Unit of Guangzhou Red Cross Hospital, Medical College, Jinan University, Tongfu Road 396, 510120 Guangzhou, China; ^4^Department of Cardiology, Sun Yat-Sen Memorial Hospital, Sun Yat-Sen University Guangzhou, Yanjiang Road, 107, 510120 Guangzhou, China

## Abstract

Persistent inflammation disrupts functional recovery after spinal cord injury (SCI). Peroxisome proliferator-activated receptor gamma (PPAR-*γ*) activation promotes functional recovery in SCI rats by inhibiting inflammatory cascades and increasing neuronal survival. We sought to clarify the relationship between PPAR-*γ* activation and NACHT, LRR and PYD domain-containing protein 3 (NLRP3) inflammasome suppression, and the role of NF-*κ*B in activating the NLRP3 inflammasome in neurons. In SCI rats, we found that rosiglitazone (PPAR-*γ* agonist) inhibited the expression of caspase-1. In *in vitro* neurons, G3335 (PPAR-*γ* antagonist) reversed the rosiglitazone-induced inhibition of caspase-1, interleukin 1 (IL-1*β*), and interleukin 6 (IL-6). Rosiglitazone inhibited the expression of NLRP3, caspase-1, IL-1*β*, and IL-6. However, the activator of NLRP3 could counteract this inhibition induced by PPAR-*γ* activation. NF-*κ*B did not participate in the process of rosiglitazone-induced inhibition of NLRP3. Consistent with our *in vitro* results, we verified that locomotor recovery of SCI rats *in vivo* was regulated via PPAR-*γ*, NLRP3, and NF-*κ*B. These results suggest that PPAR-*γ* activation exerts an anti-inflammatory effect by suppressing the NLRP3 inflammasome—but not NF-*κ*B—in neurons and that PPAR-*γ* activation is a promising therapeutic target for SCI.

## 1. Introduction

Spinal cord injury (SCI), which is a kind of high disabling injury, has gradually increased with the expansion of human activities. It leads to a loss of sensation and motor functions, as well as multiple organ dysfunctions [1]. Trauma in SCI triggers intraparenchymal inflammation and systemic immune activation, which further exacerbates neuropathology and stimulates tissue repair [2]. However, persistent inflammation after trauma disrupts functional recovery after SCI [3, 4]. The inflammasome is a multiprotein oligomer that is responsible for the activation of inflammatory responses [5]. In the central nervous system (CNS), NLRP3, the core component of the inflammasome, is involved in the generation of an innate immune inflammatory response [6]. The NLRP3 inflammasome consists of NLRP3, ASC, and procaspase-1, and cleavage of procaspase-1 into caspase-1 further activates the inflammatory cascade [5, 7]. Importantly, inflammasome activation is a potential mediator of neuroinflammation. For example, some researchers have suggested that inflammasome activation is the essential step of neuroinflammation and a key trigger for inflammation-induced neuronal death, which is called pyroptosis [8]. Additionally, another study has demonstrated that the NLRP3 inflammasome plays a pivotal function during traumatic brain injury (TBI) and SCI in the central nervous system (CNS), and targeting of the NLRP3 inflammasome can exert neuroprotection in a rat model of SCI [9].

Peroxisome proliferator-activated receptor gamma (PPAR-*γ*) is a subtype of the PPAR nuclear receptor family. The ligands of PPAR-*γ*, such as rosiglitazone and pioglitazone, are therapeutic agents for type 2 diabetes [10]. In addition to its roles in glycolipid metabolism, cell proliferation, and differentiation, PPAR-*γ* activation alleviates inflammatory responses after acute and chronic nerve injuries [11, 12]. Nuclear factor kappa-light-chain-enhancer of activated B cells (NF-*κ*B) is a classical nuclear transcription factor involved in many physiological processes, such as regulating immune responses and promoting immune cell maturation [13]. The relationship between NF-*κ*B and the NLRP3 inflammasome activation has been controversial [14–17]. Therefore, the effects of NF-*κ*B on the NLRP3 inflammasome may be cell- and pathophysiology-specific.

We have previously reported that the PPAR-*γ* ligand, rosiglitazone, promotes functional recovery after SCI by blocking the loss of local neurons and decreasing NF-*κ*B expression [18, 19]. Loss of local neurons from SCI is largely induced by persistent inflammation. Both NF-*κ*B and the NLRP3 inflammasome are pivotal for inflammation, but the effect of PPAR-*γ* either on NF-*κ*B or on the NLRP3 inflammasome remains poorly understood. In this study, we verified that PPAR-*γ* activation induces activation of NF-*κ*B and the NLRP3 inflammasome and elucidated the roles of these activations during PPAR-*γ* agonist-induced suppression of inflammation.

## 2. Materials and Methods

### 2.1. Animals

A T9-T10 laminectomy was performed in adult female Sprague–Dawley (SD) rats, and SCI was induced by dropping an impactor (10 g weight rod, 2.5 mm in diameter) from a height of 25 mm as previously reported [18] under halothane anesthesia (induction: 4%; maintenance: 2% in an oxygen and nitrous oxide (50 : 50) mixture). SCI was considered to be established—per the standard for successful rat models of SCI—if all the following manifestations presented in the rat during impactor dropping onto the spinal cord: (1) body shaking, (2) lower limbs rapidly retracting and bouncing, (3) tail lifting and quickly falling, (4) the surface of the local spinal cord quickly becoming dark purple, and (5) hindlimbs becoming completely paralyzed. All rats were housed in a temperature-controlled room at 27°C. Injured rats underwent manual bladder compression twice a day for urine excretion. Sham rats received the same operation but without impactor dropping. SCI rats were treated with rosiglitazone (3 mg/kg, Cayman), G3335 (2 mg/kg; PPAR-*γ* antagonist; Sigma), fusicoccin (dissolved in 0.5% ethanol, 100 *μ*g/ml, Sigma), or MSU (dissolved in saline, 200 *μ*g/ml, Sigma) via injection (i.p. at 5 min, 6 h, and 24 h after SCI).

The Basso-Beattie-Bresnahan locomotor rating scale (BBB scale) was used for motor function assessment. To ensure that all animals began with a score of 21, all rats were tested prior to the injury. The rats were then tested at 8 h and on days 1, 3, 7, 14, 21, and 28 postinjury (or up to the day the animal was euthanized). Each rat was scored for 4 min by two observers blinded to the study groups.

### 2.2. Immunohistochemical Staining

After surgery, rats were anesthetized by chloral hydrate (10%; 0.33 ml/kg) and sacrificed. For histological experiments, sacrificed rats were continuously perfused with 0.9% of 37°C saline and 4% paraformaldehyde. Subsequently, the spinal cord was placed in the postfixation solution for 6 h, followed by 20% sucrose buffer overnight at 4°C. Sections spanning the lesion site were immunolabeled with a caspase-1 antibody (rabbit anti-rat, Sigma, 1 : 400), and hematoxylin and eosin (HE) staining was performed on transverse sections of the spinal cord. All imaged sections of the rat spinal cord, containing the lesion, were analyzed by a blinded observer (sections were spaced 100 *μ*m apart), beginning 2.4 mm rostral to the epicenter and spanning to 2.4 mm caudal to the epicenter. A Nikon Fluorescence Microscope E800 equipped with the Spot Digital was used to capture images. Quantification of caspase-1 staining optical intensities or positive cell numbers in rat spinal cords was performed with the NIH software Image-Pro Plus 6.0.

### 2.3. Extraction and Culture of Spinal Cord-Derived Neurons

Primary spinal cord neuronal cultures were obtained from both SCI and sham rats. Rats were anesthetized and sacrificed as above, followed by spinal cord removal. The removed spinal cord was washed and cut into two to three pieces, which were digested using trypsin and collagenase. After digestion, neurons were isolated from the digested spinal cord tissue using an OptiPrep (Sigma-Aldrich) density gradient adapted from Brewer. Isolated neurons were plated in 1 ml Neurobasal-A media, supplemented with B27, GlutaMAX (all obtained from Invitrogen), and penicillin or streptomycin (Gibco, New York, America) on poly-D-lysine (Sigma-Aldrich)-coated plates at approximately 300,000 cells/well. Cells were cultured in a 37°C, 5% CO_2_ humidified atmosphere. Half of the media was replaced every two days with fresh media containing AraC (Sigma-Aldrich) to prevent astrocyte growth. The cultures were confirmed to have >90% neurons as seen by morphology on the light microscope, as well as confirmed by microtubule-associated protein 2-positive neuronal staining.

Neurons derived from the spinal cord of SCI rats were divided into the following six groups and given the following concentrations of drugs according to previous reports [18–21]: control (treated with DMSO), rosiglitazone (1 *μ*mol/l in DMSO [Abcam, ab142179]), G3335 (0.1 *μ*mol/l), rosiglitazone+MSU (rosiglitazone: 1 *μ*mol/l; MSU: 150 *μ*g/ml), fusicoccin (an NF-*κ*B activator (20 *μ*mol/l)), and PDTC (an NF-*κ*B inhibitor (25 mmol/l)).

### 2.4. Biochemical Detections

The productions of interleukin 1 (IL-1*β*) and interleukin 6 (IL-6) were determined by the enzyme-linked immunosorbent serologic assay (ELISA) using commercially available kits (GBD) according to the manufacturer's instructions, after indicated treatments.

Western blotting was used to detect caspase-1, NLRP3, and NF-*κ*B expressions. Briefly, the total cellular protein was extracted and separated by SDS-PAGE. SDS-PAGE was blotted to the PVDF membrane and incubated with anti-caspase-1 P20 (1 : 700; Abcam), anti-NF-*κ*B (1 : 400; Abcam), anti-NLRP3 (1 : 600; Santa Cruz), anti-P-I*κ*B-*α* (1 : 1000, Cell Signaling Technology), anti-procaspase-1 (1 : 800; Abcam), anti-cleaved IL-1*β* (1 : 700, Santa Cruz), and anti-IL-1*β* (full length) (1 : 1000, Abcam). The membranes were then incubated with the horseradish peroxidase-labeled secondary antibody, which was exposed to ECL color development reagents. The membranes were developed using the ChemiDoc-It™ TS2 Imaging System (Bio-Rad), and the relative optical density was analyzed using the ImageJ2x software (National Institute of Health, Bethesda, MD, USA).

### 2.5. Statistical Analysis

Normally distributed data are expressed as the mean ± SD. All statistical analyses were conducted using SPSS 14.0 software. Comparisons among groups were performed by one-way analysis of variance (ANOVA) followed by Tukey–Kramer multiple comparison post hoc tests.

## 3. Results

### 3.1. Intramedullary Hemorrhage, Cavity Formation, and Reduction of Neurons Occur during the First 28 Days after SCI in Rats

**Figure 1:**
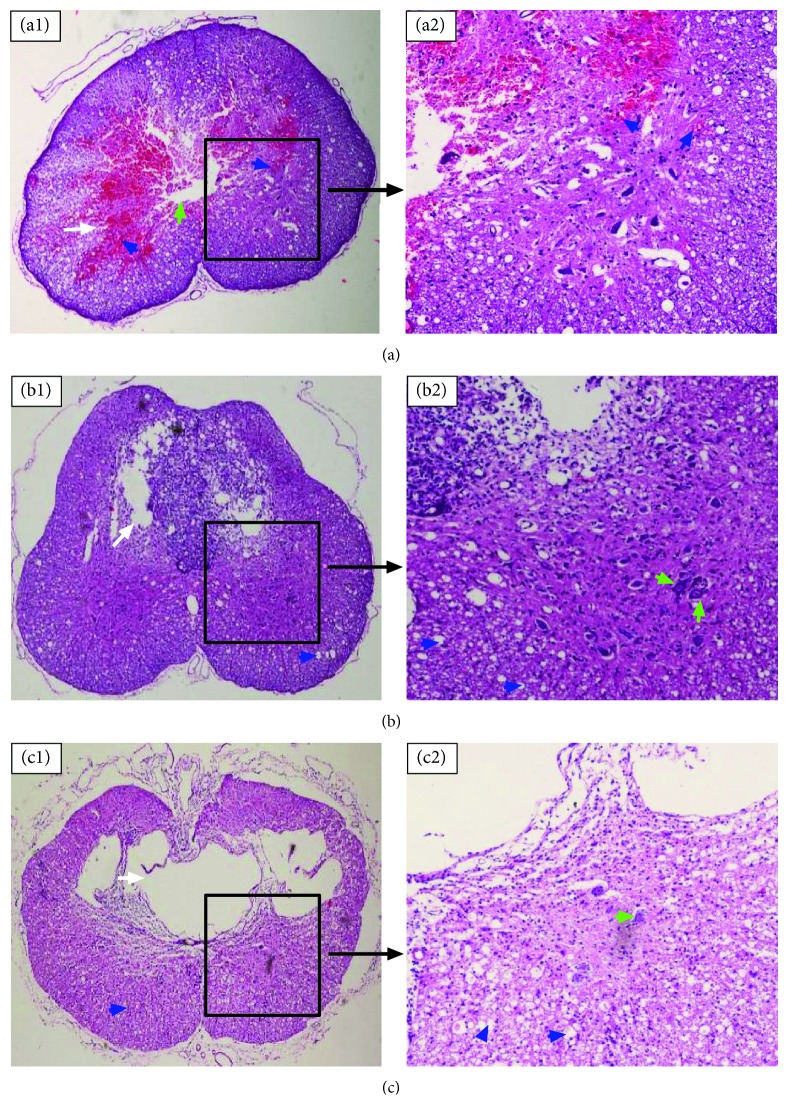
Representative HE staining of the rat spinal cord after SCI. (a1) Spinal cord hyperemia and central tube structural disorder was observed in the transverse section of the spinal cord on the 1st day after SCI (10x); (a2) in the transverse section of the ventral horn on the 1st day after SCI, red blood cells infiltrated around neurons (20x). (b1) A syringomyelia was forming in the transverse section of the spinal cord on the 7th day after SCI (10x); (b2) in the transverse section of the ventral horn on the 7th day after SCI, we observed survival of neural cell bodies and increasing gaps among myelin sheaths (20x). (c1) The syringomyelia had formed in the transverse section of the spinal cord on the 28th day after SCI (10x); (c2) in the transverse section of the ventral horn on the 28th day after SCI, we observed survival of neural cells and larger gaps among myelin sheaths (20x).

In order to observe structural changes in the spinal cord after SCI, HE staining was performed on transverse sections of the spinal cord on the 1st, 7th, and 28th days after SCI. On the 1st day, we observed spinal cord hyperemia (white arrow) (Figure 1(a1)), central tube structural disorder (green arrow) (Figure 1(a1)), and red blood cells infiltrated around neurons in the ventral horn (blue arrow) (Figures 1(a1) and 1(a2)). On the 7th day, red blood cells in the damaged area were gradually absorbed, syringomyelia was forming, and necrotic neurons were observed in the syringomyelia (white arrow) (Figure 1(b1)). We also observed red blood cell absorption and the survival of cell bodies, including neurons, in the ventral horn of the spinal cord (green arrow) (Figure 1(b2)), as well as increasing gaps among myelin sheaths (blue arrow) (Figure 1(b2)). On the 28th day, necrotic neural cells and red blood cells had been absorbed, and the syringomyelia had been formed (white arrow) (Figure 1(c1)). In the gray matter, survival of cells, including neurons, was observed (green arrow) (Figure 1(b2)). Additionally, the white matter had undergone substantial changes, and the gaps among myelin sheaths had become larger (blue arrow) (Figures 1(c1) and 1(c2)).

### 3.2. Rosiglitazone Promotes the Number of Survived Neural Cells and Inhibits Expression of Caspase-1 in the Ventral Horn of SCI Rats

**Figure 2:**
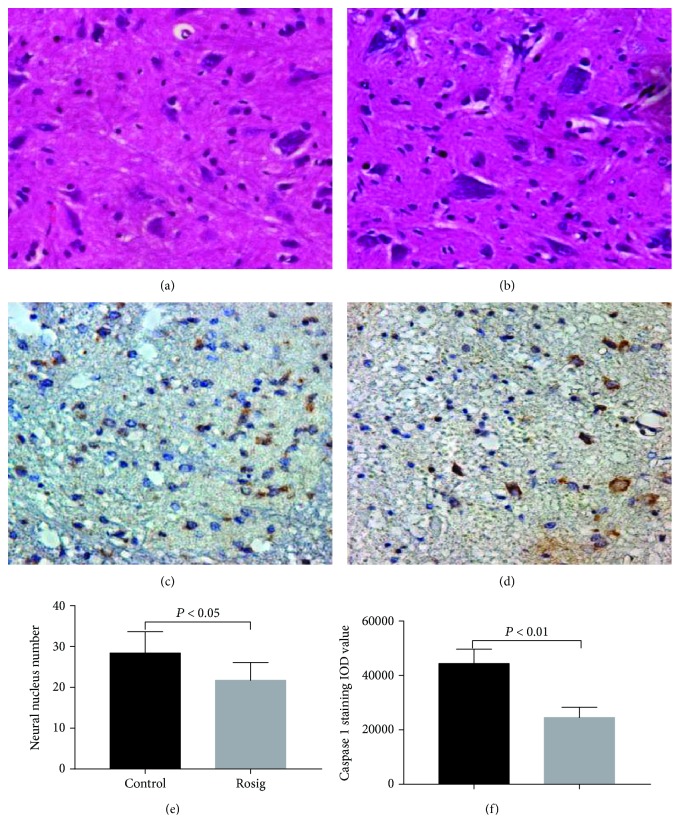
Representative HE staining in the ventral horn of the rat spinal cord and representative caspase-1 staining in the ventral horn of the rat spinal cord (40x). (a, b) Nissl bodies were visible in some neurons. (c, d) The positive staining mainly localized in the cytoplasm, but a small amount of positive staining was also observed in some nuclei (dark brown is the positive staining). (e) The number of neural nuclei in the rosiglitazone group was higher than that of the control group. (f) The expression of caspase-1 in the rosiglitazone group was lower than that in the control group. Data are expressed as mean ± SD (*n* = 6).

The number of neural nuclei in the rosiglitazone group (28.4 ± 5.23) was higher than that of the control group (21.72 ± 4.35) (*p* < 0.05, *n* = 6) (Figures 2(a), 2(b), and 2(e)). The expression of caspase-1 in the ventral horn of the spinal cord was detected by immunohistochemistry. The expression of caspase-1 was significantly lower in rosiglitazone animals (24,538 ± 3772.3) on the 7th day compared to that in the control group (44,379 ± 5273.4) (*p* < 0.01, *n* = 6) (Figures 2(c), 2(d), and 2(f)).

### 3.3. Identification of Neurons Derived from the Spinal Cord in *In Vitro* Experiments

**Figure 3:**
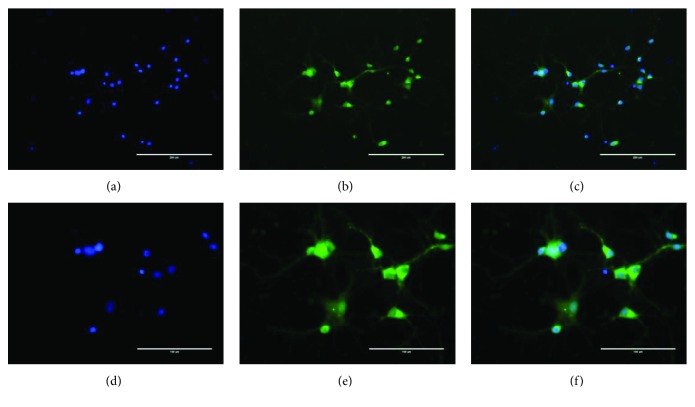
Neurons from the rat spinal cord: (a) DAPI immunofluorescent staining (20x), (b) NSE immunofluorescent staining (20x), (c) NSE-GFP-DAPI merge (20x), (d) DAPI immunofluorescent staining (40x), (e) NSE immunofluorescent staining (40x), and (f) NSE-GFP-DAPI merge (40x). Immunofluorescent staining was performed to identify neurons. NSE/DAPI > 90%. Each experiment was repeated three times.

We observed in the cell culture medium that neuronal soma was clearly visible, large, and full, exhibited a strong stereoscopic effect, had a good refractive index, and possessed a slender protrusion. Clear network connections among neurons were formed (Figures 3(a)–3(f)).

### 3.4. PPAR-*γ* Activation Inhibits Neuronal Expression of Caspase-1, IL-1*β*, and IL-6 but Does Not Affect NF-*κ*B Expression

**Figure 4:**
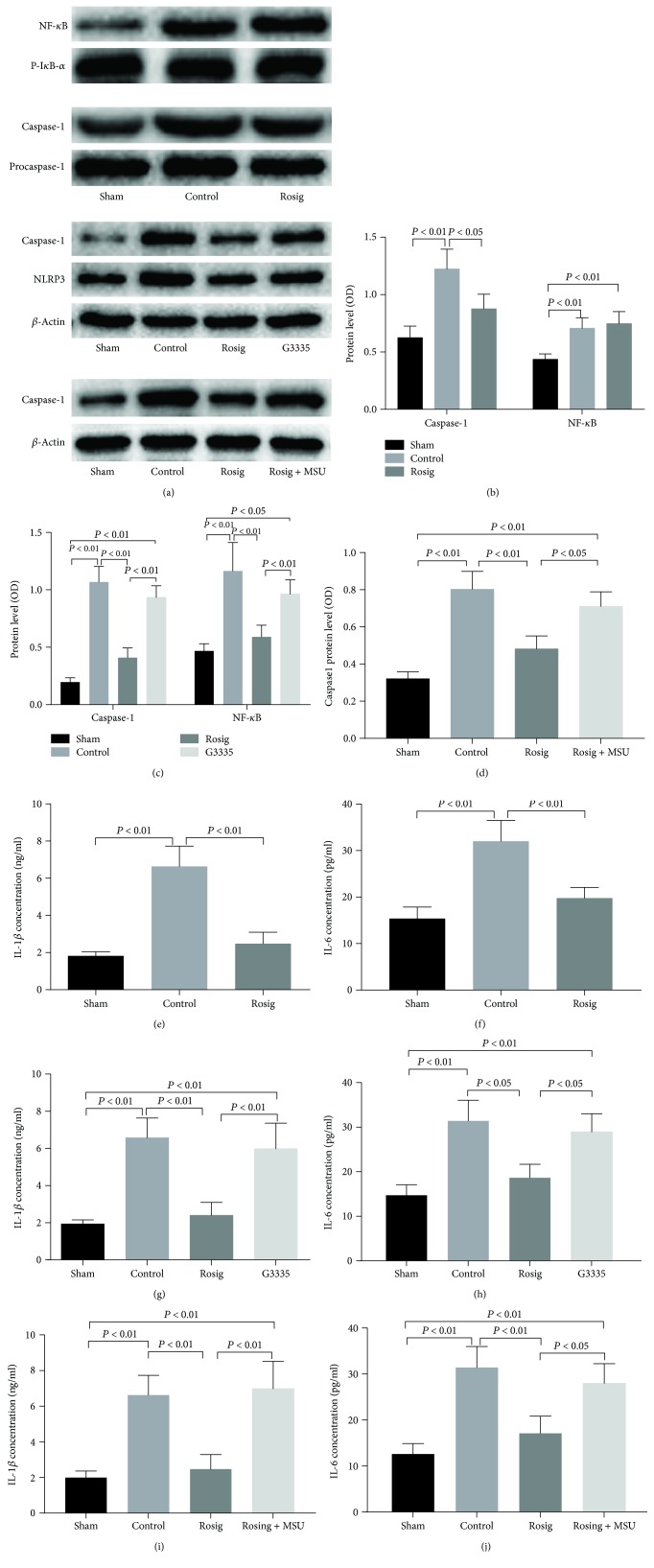
(a) Representative Western blots showing the effects of rosiglitazone on caspase-1, NF-*κ*B, and NLRP3 of sham, control, rosiglitazone, G3335, and rosiglitazone+MSU groups. (b) Relative caspase-1 and NF-*κ*B protein levels in sham, control, and rosiglitazone groups. (c) Relative caspase-1 and NLRP3 protein levels in sham, control, rosiglitazone, and G3335 groups. (d) Relative caspase-1 protein levels in sham, control, rosiglitazone, and rosiglitazone+MSU groups. (e, f) IL-1*β* and IL-6 expression levels in sham, control, and rosiglitazone groups. (g, h) IL-1*β* and IL-6 expression levels in sham, control, rosiglitazone, and G3335 groups. (i, j) IL-1*β* and IL-6 expression levels in sham, control, rosiglitazone, and rosiglitazone+MSU groups. Data are expressed as mean ± SD (*n* = 6).

In our Western blotting experiments, expression of caspase-1 in the control group was higher than that of the rosiglitazone group and sham group. The lowest expression of NF-*κ*B was found in the sham group, and there was no significant difference between the control and rosiglitazone groups (Figures 4(a) and 4(b)). In the ELISA experiments, the expression of IL-1*β* and IL-6 in the rosiglitazone group was lower than that in the control group, and there was no significant difference between the rosiglitazone and sham groups (Figures 4(e) and 4(f)). These results suggested that PPAR-*γ* activation may inhibit inflammatory cytokines *in vitro*.

### 3.5. PPAR-*γ* Antagonists Reverse the Inhibitory Effect of Rosiglitazone on Inflammatory Cytokines, and PPAR-*γ* Activation Inhibits the Expression of NLRP3

In the Western blotting experiments, the expression of caspase-1 and NLRP3 in rosiglitazone was lower than that of the control and G3335 groups. The expression of caspase-1 and NLRP3 in the rosiglitazone group was not significantly different than that of the sham group. After treatment with PPAR-*γ* antagonists, the expression of caspase-1 and NLRP3 was increased again (Figures 4(a) and 4(c)). In the ELISA experiments, the expression of IL-1*β* and IL-6 in the rosiglitazone group was lower than that of the control group. After treatment with PPAR-*γ* antagonists, the expression of IL-1*β* and IL-6 was restored, as there was no significant difference between their expressions in the rosiglitazone and sham groups (Figures 4(g) and 4(h)).

### 3.6. NLRP3 Activation Reverses the Inhibitory Effect of Rosiglitazone on Inflammatory Cytokines and Caspase-1

To elucidate the role of NLRP3 and inflammasomes in PPAR-*γ*-induced biological effects, we used MSU (the activator of NLRP3) to activate NLRP3. In Western blotting experiments, the expression of caspase-1 in the rosiglitazone group was lower than that of the control group. However, after rosiglitazone and MSU were treated simultaneously, the expression of caspase-1 was restored because there was no significant difference between its expression in the sham and rosiglitazone groups (Figures 4(a) and 4(d)).

In the ELISA experiment, the expression of IL-1*β* and IL-6 in the rosiglitazone group was lower than that of the control group. After the NLRP3 agonist MSU treatment, the expression of IL-1*β* and IL-6 was restored because there was no significant difference between their expressions in the sham and rosiglitazone groups (Figures 4(i) and 4(j)).

### 3.7. NF-*κ*B Is a Negative Regulator of Neuronal IL-1*β* Secretion but Has No Effect on Neuronal Expression of Caspase-1 and NLRP3

**Figure 5:**
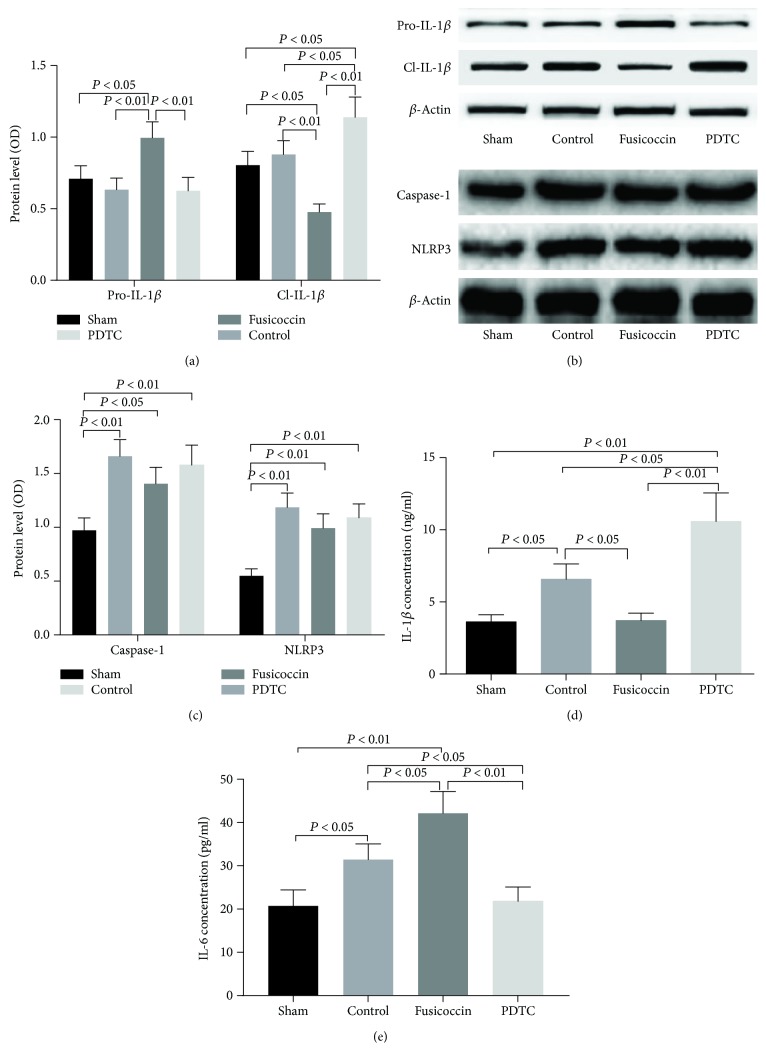
The protein expression of caspase-1 and NLRP3 and the concentrations of IL-1*β* and IL-6 in sham, control, fusicoccin, and PDTC groups: (a) relative pro-IL-1*β* and Cl-IL-1*β* protein levels; (b) representative Western blots of pro-IL-1*β* and Cl-IL-1*β*. Representative Western blots showing the effects of NF-*κ*B on caspase-1 and NLRP3. (c) Relative caspase-1 and NLRP3 protein levels; (d) IL-1*β* expression level; (e) IL-6 expression level. Data are expressed as mean ± SD (*n* = 6).

In the Western blotting experiments, there was no significant difference among the control, fusicoccin, and PDTC groups in the expression of caspase-1 or NLRP3. The lowest expression of caspase-1 and NLRP3 was found in the sham group (Figures 5(b) and 5(d)). In the ELISA experiment, the expression of IL-1*β* in the NF-*κ*B activator (fusicoccin) group was lower than that of the control group, and after treatment with the NF-*κ*B inhibitor, PDTC, the expression of IL-1*β* was higher than that of the other three groups. The expression of IL-6 in the fusicoccin group was higher than that of the other three groups (Figures 5(a) and 5(c)).

### 3.8. PPAR-*γ* Activation Is the Most Effective in Promoting Locomotor Recovery after SCI in Rats

All rats had a BBB score of 21 before SCI and lost locomotor function 8 h after SCI.

**Figure 6:**
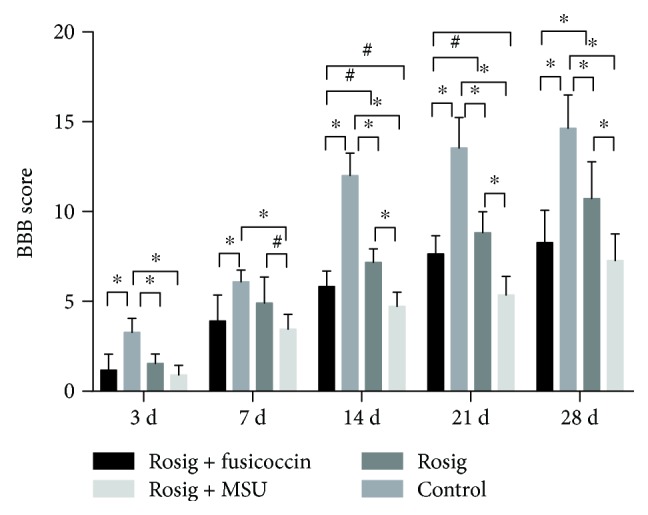
BBB scores after SCI. Data are expressed as mean ± SD (*n* = 11). ^∗^*p* < 0.01, ^#^*p* < 0.05. One-way ANOVA followed by a Tukey–Kramer multiple comparison post hoc test.

At the 24 h time point, the BBB scores of all the groups were still not >2. On the 3rd day, BBB scores in the rosiglitazone group were higher than those of the other three groups (*p* < 0.05, *n* = 11). On the 7th day, the rosiglitazone group showed significantly higher BBB scores than those of the control group and rosiglitazone+MSU group (*P* < 0.05, *n* = 11). There was no statistical difference between the rosiglitazone group and rosiglitazone+fusicoccin group. On the 14th, 21st, and 28th days, the rosiglitazone group showed significantly higher BBB scores than the other three groups (*p* < 0.05, *n* = 11). And the rosiglitazone+fusicoccin group showed significantly higher BBB scores than those of the rosiglitazone+MSU group (*p* < 0.05, *n* = 11) (Figure 6).

## 4. Discussion

Neuronal survival is a basis for functional recovery after SCI [22]. In this study, early hemorrhage and later syringomyelia formation were observed in SCI rats. The number of neural cells decreased in the ventral horn, while a gradually loose structure in the white matter area presented during this process. SCI frequently induces neuronal death by secondary inflammation [23]. Therefore, blocking persistent inflammation is a key to treating SCI. Rosiglitazone, a ligand of PPAR-*γ*, inhibits CNS inflammation [24]. In this study, we observed increased survival of Nissl-stained soma and nuclei in the ventral horn after rosiglitazone treatment in SCI rats.

NLRP1 and NLRP3 inflammasomes play a pivotal function in SCI [8]. It has been demonstrated that SCI stimulates a complex scenario of inflammasome activation at the injured site and that SDF-1a-mediated neuroprotection presumably depends on the attenuation of the inflammasome complex [9]. Some researchers have reported that rat spinal cord neurons contain a caspase-1, pro-IL*β*, and pro-IL-18 activating complex which is different from the human NALP1 inflammasome that constitutes an important arm of the innate CNS inflammatory response after human SCI [25]. The activation of the inflammasome in neurons is important for functional recovery after SCI. For example, it has been demonstrated that P2X4 knock-out mice show impaired inflammasome signaling in the spinal cord, resulting in significant tissue sparing and improvement of functional outcomes [6]. Additionally, the same study has indicated that P2X4 receptors influence inflammasome signaling, which is involved in caspase-1 activation and IL-1*β* processing in neurons after SCI [6]. It has also been reported that HO-1 protects spinal cord neurons after SCI through inhibiting NLRP1 inflammasome formation [26]. In this study, the reduced expression of NLRP3 and caspase-1 was observed in spinal cord-derived neurons after rosiglitazone treatment and was counteracted by the PPAR-*γ* inhibitor, G3335.

The NLRP3 inflammasome is a multiprotein complex composed of NLRP3, ASC, and procaspase-1. The inflammasome can mediate the activation of caspase-1, which is a self-evolving protease. Pro-IL-1 is cleaved by recombinant caspase-1 *in vitro*. Moreover, activated IL-1*β* and IL-18 induce inflammatory cascades [27]. Therefore, caspase-1 activation is a key signal for NLRP3 inflammasome activation. In this study, the expression of caspase-1 was inhibited after rosiglitazone treatment, and the expression of caspase-1 was elevated when PPAR-*γ* was blocked. This suggests that PPAR-*γ* activation is associated with NLRP3 inhibition in spinal cord-derived neurons.

PPAR-*γ* is a negative regulator of NLRP3 inflammasome activation. For example, Song et al. [28] have demonstrated that the PPAR-*γ* agonist, astragaloside IV, ameliorates neuroinflammation-induced depressive-like behaviors in mice via the PPAR-*γ*/NF-*κ*B/NLRP3 inflammasome axis. Wang et al. [29] have suggested that umbelliferone ameliorates cerebral ischemia-reperfusion injury via upregulating PPAR-*γ* expression and suppressing the TXNIP/NLRP3 inflammasome. Kumar et al. [30] have considered that the PPAR-*γ* ligand, 15-lipoxygenase, metabolites of *α*-linolenic acid (13-(S)-HPOTrE and 13-(S)-HOTrE), mediates anti-inflammatory effects by inactivating the NLRP3 inflammasome. In this study, decreased NLRP3 expression was observed after rosiglitazone treatment, and the expression of NLRP3 was increased when PPAR-*γ* was inhibited. These results were consistent with the change in caspase-1. MSU crystals could induce the assembly of NLRP3 inflammasomes to activate caspase-1. Since PPAR-*γ* activation leads to suppression of the inflammasome, we performed rescue experiments, using MUS, to activate NLRP3 and to restore activity of the inflammasome via inflammasome agonism. We found that MSU treatment could restore the rosiglitazone-induced inhibition of caspase-1, IL-6, and IL-1*β*. These results suggest that the anti-inflammatory actions of rosiglitazone work through inhibiting the NLRP3 inflammasome.

As reported previously, NF-*κ*B and MAPK signaling promote NLRP inflammasome activation in neurons following ischemic stroke [15]. However, some researchers have found that NF-*κ*B is a negative regulator for the activation of the NLRP3 inflammasome [16, 31]. The different responses of the inflammasome to NF-*κ*B might depend on cell type identity. In this study, PPAR-*γ* activation had little effect on the changes of NF-*κ*B in neurons. Furthermore, either increases or decreases of NF-*κ*B had no effect on NLRP3 or caspase-1. Interestingly, IL-1*β* was negatively regulated by NF-*κ*B, while IL-6 was positively regulated by NF-*κ*B. This result suggests that besides the NLRP3 inflammasome pathway, NF-*κ*B may regulate inflammation via another pathway. Our results are consistent with those reported by Greten et al., who have demonstrated that NF-*κ*B is a negative regulator of IL-1*β* secretion in myeloid cells of mice [32]. The mechanism of NF-*κ*B on inflammation in neurons still requires further investigation.

The effects of rosiglitazone on accelerating functional recovery after SCI in rats have been demonstrated [33, 34]. Interestingly, in addition to rosiglitazone, which is recognized as an effective treatment for locomotor recovery in SCI rats, this study found that rosiglitazone plus an NF-*κ*B activator (fusicoccin) was superior to the treatment combining rosiglitazone and an NLRP3 agonist (MSU) for the locomotor recovery at 14, 21, and 28 days after SCI. This may be due to the complexity of the spinal microenvironment after trauma. Indeed, different responses of the NLRP3 inflammasome to NF-*κ*B highly depend on different microenvironments and cell types. Additionally, as stated above, NF-*κ*B may participate in inflammation after SCI through another pathway. This warrants further investigation, such as regarding the role of NF-*κ*B in NLRP3 inflammasome activation in microglia.

## 5. Conclusion

Persistent inflammation hinders functional recovery after spinal cord injury. We suggest that PPAR-*γ* activation exerts an anti-inflammatory effect by suppressing the NLRP3 inflammasome—but not NF-*κ*B—in neurons. Hence, PPAR-*γ* activation may be a promising therapeutic strategy for the treatment of SCI.

## Data Availability

The data used to support the findings of this study are available from the corresponding author upon request.
